# Antifungal Activity of Polyoxometalate-Ionic Liquids on Historical Brick

**DOI:** 10.3390/molecules25235663

**Published:** 2020-12-01

**Authors:** Katarzyna Rajkowska, Anna Koziróg, Anna Otlewska, Małgorzata Piotrowska, Elena Atrián-Blasco, Isabel Franco-Castillo, Scott G. Mitchell

**Affiliations:** 1 Institute of Fermentation Technology and Microbiology, Lodz University of Technology, Wólczańska 171/173, 90-924 Lodz, Poland; anna.kozirog@p.lodz.pl (A.K.); anna.otlewska@p.lodz.pl (A.O.); malgorzata.piotrowska@p.lodz.pl (M.P.); 2 Instituto de Nanociencia y Materiales de Aragón (INMA), Consejo Superior de Investigaciones Científicas-Universidad de Zaragoza, 50009 Zaragoza, Spain; elenaab@unizar.es (E.A.-B.); isabelfrancocastillo@gmail.com (I.F.-C.); 3 Center for Biomedical Research Network-Bioengineering, Biomaterials and Nanomedicine (CIBER-BBN), Instituto de Salud Carlos III, 28029 Madrid, Spain

**Keywords:** polyoxometalate ionic liquids, antifungal activity, historical brick, biodeterioration, environmental scanning transmission electron microscopy

## Abstract

Moulds inhabiting mineral-based materials may cause their biodeterioration, contributing to inestimable losses, especially in the case of cultural heritage objects and architectures. Fungi in mouldy buildings may also pose a threat to human health and constitute the main etiological factor in building related illnesses. In this context, research into novel compounds with antifungal activity is of high importance. The aim of this study was to evaluate the antifungal activity of polyoxometalate-ionic liquids (POM-ILs) and their use in the eradication of moulds from historical brick. In the disc diffusion assay, all the tested POM-ILs inhibited growth of a mixed culture of moulds including *Engyodontium album*, *Cladosporium cladosporioides*, *Alternaria alternata* and *Aspergillus fumigatus*. These were isolated from the surfaces of historical brick barracks at the Auschwitz II-Birkenau State Museum in Oświęcim, Poland. POM-IL coatings on historical brick samples, under model conditions, showed that two compounds demonstrated very high antifungal activity, completely limiting mould growth and development. The antifungal activity of the POM-ILs appeared to stem from their toxic effects on conidia, as evidenced by environmental scanning transmission electron microscopy observations. The results herein indicated that POM-ILs are promising disinfectant materials for use not only on historical objects, but probably also on other mineral-based materials.

## 1. Introduction

The susceptibility of brick, concrete, mortar, stone and other mineral-based building materials to colonisation by various organisms is defined as bioreceptivity. This concept was introduced by Guillette [1] and included chemical and physical parameters such as mineralogical composition, water availability, temperature, pH, surface porosity and roughness influence on biological succession [2,3]. From a biological point of view, mineral-based building materials represent an extreme microniche for microbial growth due to large variations in environmental parameters and higher desiccation conditions in vertical and subvertical surfaces [3]. Bacteria and fungi inhabit pits, cracks and fissures on the surface of mineral-based materials, and they often may interact with substrate causing their deterioration [4]. Microorganisms, especially fungi, as a result of metabolic activity, secrete different corrosive organic acids (e.g., oxalic, citric, formic, fumaric, gluconic acid), which lead to increased solubilisation of material components in water and consequently to structural damage. Moreover, the mineral matrix structure may be mechanically destroyed by the penetrative growth of fungal hyphae [5]. *Aspergillus* spp. (*A. fumigatus*, *A. versicolor*, *A. niger*), *Penicillium* spp. (*P. chrysogenum*, *P. frequentans*, *P. simplissimum*), *Cladosporium herbarum*, *Fusarium oxysporum*, *Mucor hiemalis*, *Alternaria alternata* and *Rhizopus oryzae* are important deteriogenic fungi known to flourish on stone materials [5,6]. Nevertheless, the literature focusing on microbial colonisation of mineral-based building materials, especially brick, is very limited.

It is important to note that fungi-induced biodeterioration is observed not only on cultural heritage architectures and artefacts, but also on modern buildings, museums, storerooms and a variety of other materials [6,7,8]. This deteriorative phenomenon is also problematic from a health and safety perspective, because the excessive development of moulds in damp building materials and poorly ventilated buildings means that the resulting air contamination poses a serious risk factor for the health of building users [9]. Although moulds rarely cause serious diseases in healthy individuals, mostly affecting only those with weakened immune systems, however, continuous living in damp and mouldy buildings can have detrimental long-term effects in healthy people and is associated with an increased risk of developing asthma, rhinitis, allergies, dyspnoea, wheeze, cough, hypersensitivity pneumonitis, superficial and systemic infections (mycoses) and mycotoxicosis [9,10,11]. In the human body, moulds elicit an immune response through cytotoxic effects and the production of mediators of inflammatory reactions. Allergic reactions to *Alternaria*, *Cladosporium*, *Aspergillus*, *Penicillium* and *Fusarium* are the most often clinically detected allergies [12,13]. Moreover, *Aspergillus*, *Penicillium* and *Stachybotrys* genera produce mycotoxins, which can form aerosols and enter the human body through inhalation along with fungal spores. Mycotoxins are compounds with mutagenic, teratogenic, carcinogenic, dermatotoxic, hepatotoxic, neurotoxic properties [14,15]. Furthermore, it has been demonstrated that moulds play a major role in Sick Building Syndrome (SBS) or chronic fatigue syndrome [11], which is characterised by non-specific symptoms such as fatigue, nausea, drowsiness, headache, dizziness, irritability, concentration and memory disorders, as well as asthma-like symptoms [9,15]. In such cases, spores and fragments of mycelium hyphae serve as carriers of allergenic proteins and mycotoxins, nano-particulates, structural components of cells, as well as enzymes or volatile metabolites that are secreted into the environment [13,16]. 

Polyoxometalates (POMs) are an important and diverse class of nanoscale molecular metal oxides, which are characterised by a wide and versatile range of physicochemical properties that can be tuned on the molecular level [17]. Their redox properties and high oxidation activity also make them useful to a wide variety of applications, from catalysis [18] to medicine [19]. POMs cross lipid membranes, interact with proteins and are producers of reactive oxygen species, so they also display important antibacterial activity [20]. Furthermore, polyoxometalate-ionic liquids (POM-ILs), where molecular POM anions are fused with bulky and functional organic cations, e.g., organic ammonium or phosphonium cations, often result in room-temperature ionic liquids. Ionic liquids (ILs) are salts with a melting point <100 °C, whose structure can be tuned to access physicochemical properties such as wide electrochemical window, high thermal stability and null vapour pressure [21,22,23]. Importantly, unique properties of POM-ILs have been harnessed for universal pollutant removal [24] and reactive surface protecting anti-corrosion coatings [25,26,27] because both the anion and cation can be tailored to target biological, organic and inorganic pollutants including microbes, acids and reactive oxygen species [28]. Their rheological properties and hydrophobicity can also be tuned by chemical design, which are important to obtain surface coatings. Prototype POM-ILs have demonstrated excellent biofilm and corrosion protection on natural stones [27], but the broad-spectrum antimicrobial activity of these materials against different bacteria, yeast and moulds still needs to be evaluated.

The aim of this study was to assess the antifungal activity of POM-ILs and their usefulness in eradication of moulds inhabiting historical brick. Although antibacterial effects of water insoluble POM-ILs have been reported previously [26,27], however, to the best of our knowledge, this is the first study to investigate their antifungal activity and application in brick conservation.

## 2. Results and Discussion

A total of eight silicotungstate and phosphotungstate-based POM-ILs were synthesized based on previously reported experimental procedures. Please refer to Supplementary Materials for further details on synthesis and Tables S1–S4 for characterisation data. Table 1 provides a summary of the eight different POM-ILs employed in this study.

A disc diffusion method was used to assess the in vitro antifungal activity of eight different but closely related POM-ILs and their capacity to prevent or inhibit mould growth on historical brick samples. The antifungal activity was examined against a mixed culture of moulds consisting of *Engyodontium album*, *Cladosporium cladosporioides*, *Alternaria alternata* and *Aspergillus fumigatus*, that were isolated from the surfaces of historical brick barracks at the Auschwitz II-Birkenau State Museum in Oświęcim, Poland. The use of mixed culture of moulds is especially important since masonry building parts are usually inhabited not by one single strain but by a mixture of microorganisms [29]. Moreover, the environmental strains which were isolated from historical brick objects were adapted to nutrient-poor materials. 

**Figure 1:**
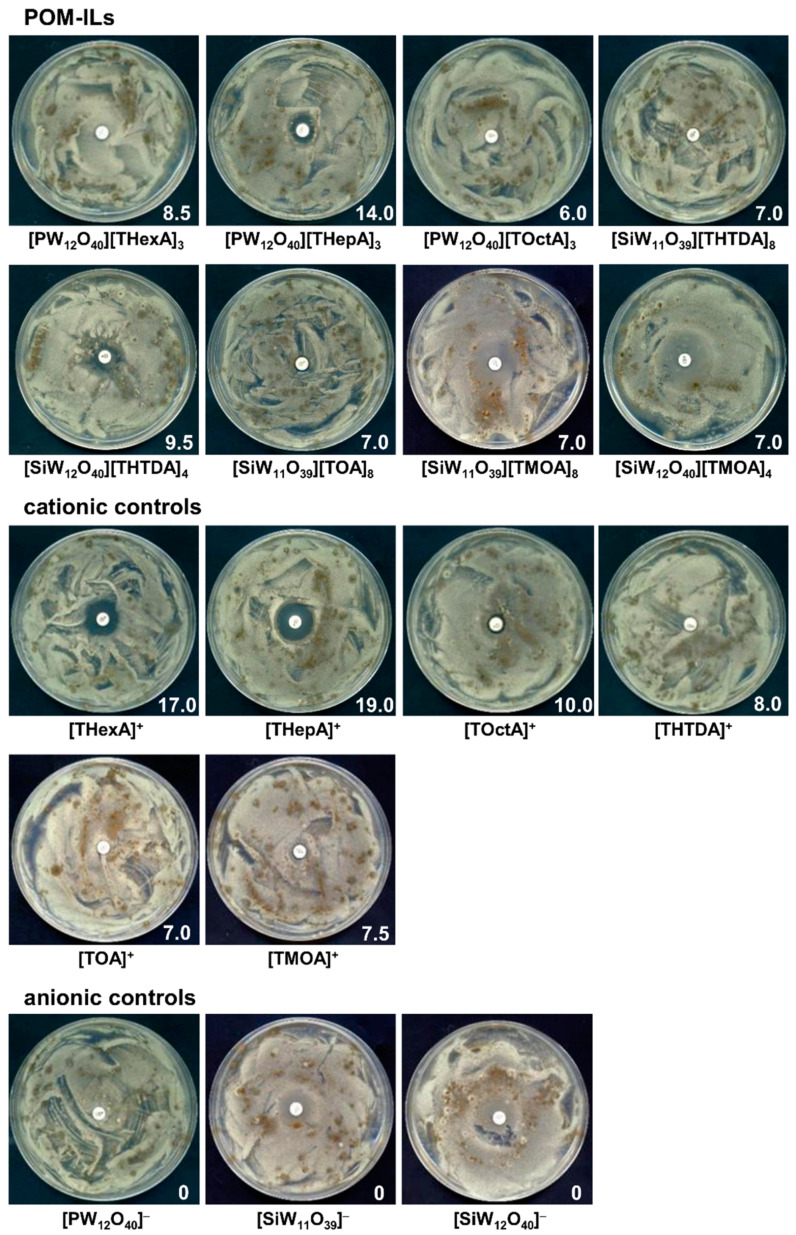
Zones of inhibition of mould growth by disc-diffusion method; the zone’s diameter (mm) is shown in white in the lower right corner of each image.

In the disc diffusion assay, all the tested POM-ILs inhibited mould growth, despite their poor aqueous solubility, although clear differences in diameters of inhibition growth zones were observed (Figure 1). The highest antifungal activity was exhibited by [PW_12_O_40_][THepA]_3_, [SiW_12_O_40_][THTDA]_4_ and [PW_12_O_40_][THexA]_3_. Other compounds acted only when the disc was in direct contact with the mould cultures, and then the growth inhibition zones equalled 6 mm. None of the anionic controls of POM-ILs showed activity against moulds, while cationic controls were highly active. The highest activity was found for [THepA]^+^ and [THexA]^+^ (Figure 1). These results indicate that diffusion-based antifungal activity of POM-ILs resulted mostly from activity of the cation species, which is consistent with previous findings of Kubo et al. [26], who explained that the antibacterial activity of these compounds arises primarily from the cationic action. 

While the diffusion method is a standard method for evaluating antimicrobial compounds and materials, however, its principal limitation is that the assay requires the diffusion of compounds out of the discs and into the agar nutrient medium. In the case of hydrophobic compounds and water insoluble compounds, their diffusion and delivery to microbial cells in aqueous media may be hampered [30,31]. The POM-ILs in this report are characterised by high viscosity and water immiscibility [27].

**Figure 2:**
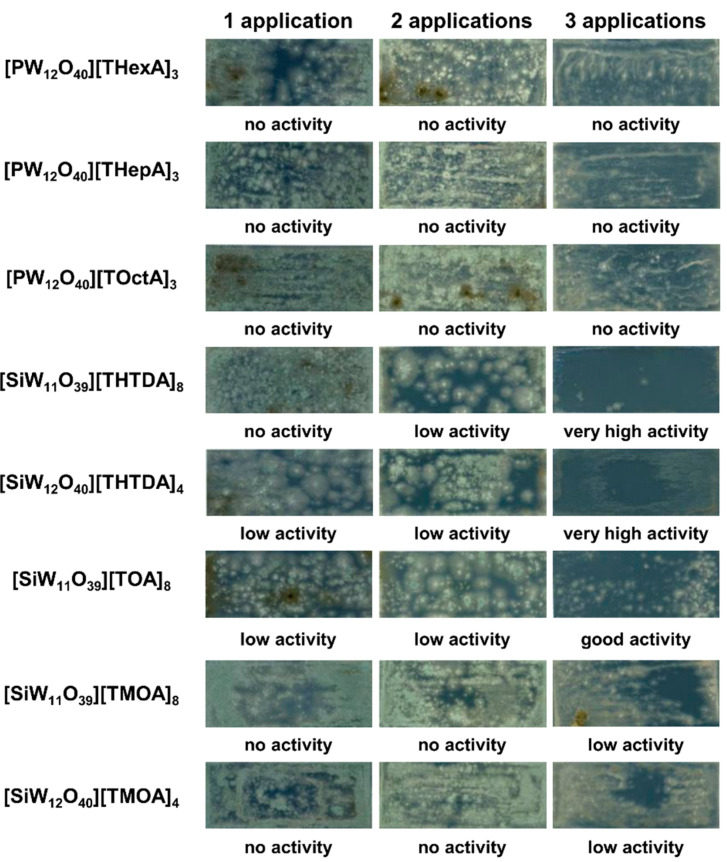
Antifungal activity of POM-ILs on brick samples inhabited by mixed culture of moulds.

Considering the water immiscibility and high viscosity of these POM-ILs, their antifungal activity was also determined on historical brick samples under model conditions with high mould content (>10^6^ cfu/cm^2^). Briefly, 19th century brick samples were inoculated with moulds and allowed to colonise the brick surface in a climate chamber (80% relative humidity and 28 °C) for three weeks. Afterwards, POM-ILs were spray-coated up to three times and the number of microorganisms after successive POM-IL applications was determined by the contact plate method whereby the antifungal activity of POM-ILs was evaluated according to a calibration scale (please refer to Materials and Methods figure). In contrast to the disc diffusion studies, the historical brick studies demonstrated that two compounds, [SiW_11_O_39_][THTDA]_8_ and [SiW_12_O_40_][THTDA]_4_, showed very high antifungal activity after three successive applications (according to our calibration scale), by completely inhibiting the growth of moulds (Figure 2). Good activity was also exhibited by [SiW_11_O_39_][TOA]_8_ after the third application, where the brick surface contamination by fungi was reduced approximately by 50%. Mould growth inhibition was also found in other POM-ILs, i.e., [SiW_11_O_39_][TMOA]_8_ and [SiW_12_O_40_][TMOA]_4_. The results indicate that POM-ILs, especially [SiW_11_O_39_][THTDA]_8_ and [SiW_12_O_40_][THTDA]_4_, can be effectively used in disinfection of historical brick, although the following key issues should be considered.

In this study, POM-ILs solutions were applied by spraying and this method was chosen to minimise the risk of mechanical removal of fungal mycelium from the surface of bricks. Consequently, this method can result in a heterogeneous coverage of the compounds on the brick surface. For this reason, if POM-ILs are to be used as protective antimicrobial coatings, it is preferable to use the brush-coating method to protect the surface of materials against the development of microorganisms, as has been reported for POM-IL coatings on natural stones [27]. 

Other important factors determining the activity of biocides are their concentration, the number of applications, type of material and, what is particularly important, the intensity of microbial growth already present on the material surface. Only in objects heavily contaminated by microorganisms and in favourable environmental conditions, macroscopic signs of microbial growth are visible and the number of moulds exceeds 10^6^ cfu/cm^2^, as is seen in this study. In general, for historical objects, fungi were detected at a lower level of 10^1^–10^3^ cfu/100 cm^2^ [32], and on building materials characterised by spore presence and active mould growth, the fungal contamination is estimated at 10^4^–10^5^ cfu/m^2^ [33]. Therefore, the real-world use of POM-ILs as antifungal agents in situ is expected to be more effective than under the more extreme model conditions reported here.

In this context, the results obtained for the tested POM-ILs are very promising in the aspect of effective use on masonry surfaces of buildings, including historical objects. In addition to the potent antimicrobial activity of the quaternary ammonium cations that largely affected the conidia plasma membranes, we hypothesised that POM-IL coatings interfere with electron transfer and respiration processes and the overall applicability and potential as protective surface coatings are also closely related to the viscosity and hydrophobicity of the material. To further analyse the antifungal activity of the most active POM-ILs from this study, environmental scanning transmission electron microscopy (ESTEM) was used to study the effect of [SiW_11_O_39_][THTDA]_8_ and [SiW_12_O_40_][THTDA]_4_ POM-ILs on fungal mycelium and conidia.

**Figure 3:**
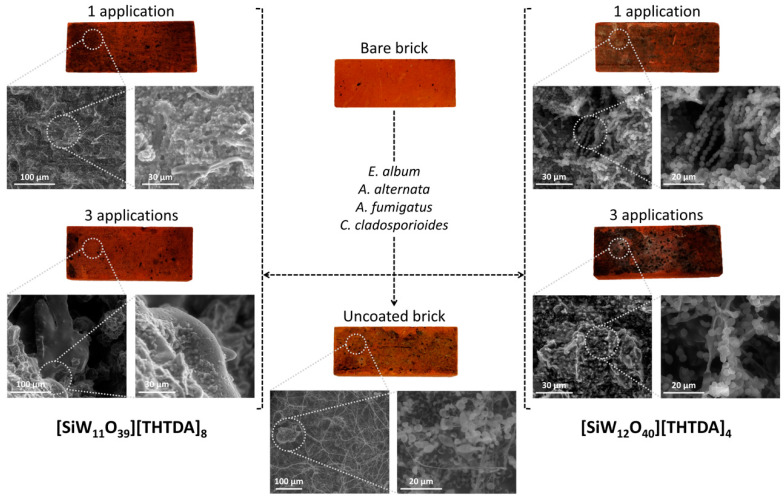
Environmental scanning transmission electron microscopy (ESTEM) images of antifungal activity of [SiW_11_O_39_][THTDA]_8_ and [SiW_12_O_40_][THTDA]_4_ POM-ILs on historical brick samples.

After the first application, solutions not only covered the mycelium and conidia, but also penetrated deep into the spatial moulds structure. After three applications, POM-ILs homogeneously covered the surface of the material together with the developed mycelium (Figure 3). ESTEM observations indicated toxic effects of [SiW_11_O_39_][THTDA]_8_ and [SiW_12_O_40_][THTDA]_4_ on fungal conidia (Figure 3 and Figure S1). In control images, conidia were collapsed as a result of moderate dehydration and a loss of turgor pressure under the ESTEM sample collection conditions. Such morphological changes are typical for conidia with intact membranes [34]. After the POM-ILs application, conidia were generally spherical, which is indicative of membrane damage and collapse. Similar results have been published previously for quaternary phosphonium ionic liquids, the mechanism of action of which are involved in damaging conidia plasma membrane [35].

The results presented herein for POM-ILs are consistent with those reported for ionic liquids; this reinforces the theorem on the key role of cation species in antifungal activity. The antimicrobial mechanism of ionic liquids results from their amphiphilic character allows their insertion into the phospholipid bilayer of plasma membranes [36]. These interactions lead to plasma membrane damage, leakage of intracellular content, and, in consequence, to cell death [37]. In moulds, substantial damages to conidia and hyphae provoked by long alkyl chains were also documented [38]. The severe ultrastructural changes in cells were associated with membrane destruction, distending of cell wall structure, formation of residual organelles in the form of membranous structures and multivesicular bodies. On the other hand, the high negative charge of polyoxometalates means that they do not readily penetrate through cytoplasmic membranes, but they can cause impairments of vital cell functions in bacteria by interaction with the proteins involved in signal-transducing systems [20]. Furthermore, POMs can interfere with bacterial electron transfer, thereby affecting respiration processes [39]. The combination of two active components in the POM-IL molecule is clearly an important antimicrobial activity enhancing property, however, the exact antifungal mode of action of POMs is still not understood and will require further detailed mechanistic experimentation. 

## 3. Materials and Methods

### 3.1. Synthesis and Characterisation of POM-ILs

POM-ILs were synthesised according to previously reported synthetic procedures [26,27]. Briefly, a desired amount of POM was dissolved in 50 mL of pre-heated distilled water at 50 °C. The mixture was stirred until complete dissolution. In a beaker, the appropriate amount of the corresponding ionic liquid was dissolved in 80 mL of the solvent and stirred until complete dissolution. Then, the POM solution was slowly added to the IL solution and the mixture was stirred for 3 h at room temperature. After this time, the mixture was transferred to a separation funnel and once the two phases were clearly separated, the organic phase—including the POM-IL—was extracted into a round bottom flask. The organic solvent was removed in a rotatory evaporator, and the product was isolated in the form of powder or viscous liquid at room temperature. 

All POM-ILs were characterised by thermogravimetric analysis (TGA Q5000, TA Instruments–Waters Corporation, New Castle, DE, USA), elemental analysis (Thermo Flash 1120, Thermo Fisher Scientific Inc., Waltham, MA, USA) and Fourier transform infrared spectroscopy (FT-IR JASCO FT/IR- 4100, Jasco Analitica Spain, Madrid, Spain). Please refer to Supplementary Materials Tables S1–S4 for characterisation data. 

### 3.2. Assessment of Antifungal Activity of POM-ILs by Disc Diffusion Method

In the assay, four mould strains in a mixed culture were used, i.e., *Engydontium album* LOCK 0590, *Cladosporium cladosporioides* LOCK 0592, *Alternaria alternata* LOCK 0594 and *Aspergillus fumigatus* LOCK 0596. All moulds were isolated from the surfaces of historical brick barracks at the Auschwitz II-Birkenau State Museum in Oświęcim, Poland. Antifungal activity was determined for POM-ILs solutions in dimethyl sulfoxide (DMSO) at a concentration of 5 mg/mL, by the disc diffusion method. Briefly, after spreading 100 µL of mixed culture of moulds in sterile saline (10^6^ conidia/mL) on malt extract agar (MEA, Merck KGaA, Darmstadt, Germany), paper discs with 20 µL of POM-ILs were placed on the agar surface. DMSO (100%) served as a control. Plates were incubated for up to 5 days at 28 °C. The results were expressed as diameters of growth inhibition zones including the diameter of the disc (Ø 6 mm).

### 3.3. Evaluation of Antifungal Activity on Brick Samples

**Figure 4:**
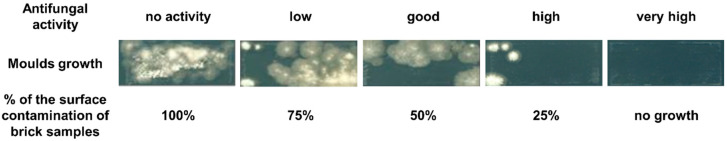
The scale of assessment of antifungal activity exhibited by POM-ILs.

Antifungal activity of POM-ILs was assessed on historical brick under model conditions, as described previously [40]. One millilitre of mixed culture of moulds (10^6^ conidia/mL) in the medium ((NH_4_)_2_SO_4_ 0.075%, K_2_HPO_4_ 0.025%, MgSO_4_·7H_2_O 0.125%, yeast extract 0.125%, glucose 0.5%, agar 0.1%, pH 6.0) was applied on the surface of brick. Brick samples (50 × 20 × 10 mm^3^) came from the demolition of a 19th century residential building. The samples inoculated with moulds were incubated in a constant climate chamber with a relative humidity of 80% for 21 days at 28 °C. Afterwards, POM-ILs were applied up to three times at an interval of 24 h, using the spraying method. The number of microorganisms after successive applications of POM-ILs was determined by the contact plate method in MEA medium. Plates were incubated for 5 days at 28 °C. The antifungal activity of POM-ILs was evaluated according to a calibration scale (Figure 4).

### 3.4. Environmental Scanning Electron Microscopy (ESEM)

Data were collected on a Quanta FEG-250 (FEI Company, Hillsboro, OR, USA) field emission SEM for high-resolution imaging working in ESEM mode, using a gaseous secondary electron detector (GSED) detector under high relative humidity conditions.

## 4. Conclusions

The tested POM-ILs as part of this project showed high antifungal activity against a mixed culture of moulds that are well-adapted to the extreme conditions of mineral-based building materials. The inhibition of mould growth by POM-ILs occurs through toxic effects on conidia, and by coating the surface of the brick material, which may consequently limit the access to oxygen and nutrients. Although the antimicrobial activity of POM-ILs was mostly associated with cations, the combination of two components in the molecule gives them unique properties such as water immiscibility and creates the opportunity to design compounds with the desired characteristics for reducing microbially induced biodeterioration.

As a result of these promising features, POM-ILs could be used as antifungal agents in a variety of applications, not limited to historical brick, as described in this current study. Furthermore, the use of POM-ILs to disinfect or form protective coatings on other mineral-based building materials or historical objects, as well as in residential buildings can also be envisaged.

## Figures and Tables

**Table 1:** Summary of the POM-ILs.

POM-IL	POM-IL Short Name
[PW_12_O_40_][(C_6_H_13_)_4_N]_3_	[PW_12_O_40_][THexA]_3_
[PW_12_O_40_][(C_7_H_15_)_4_N]_3_	[PW_12_O_40_][THepA]_3_
[PW_12_O_40_][(C_8_H_17_)_4_N]_3_	[PW_12_O_40_][TOctA]_3_
[SiW_11_O_39_][(C_6_H_13_)_3_(C_14_H_29_)N]_8_	[SiW_11_O_39_][THTDA]_8_
[SiW_12_O_40_][(C_6_H_13_)_3_(C_14_H_29_)N]_4_	[SiW_12_O_40_][THTDA]_4_
[SiW_11_O_39_][(C_8_H_17_)_4_N]_8_	[SiW_11_O_39_][TOctA]_8_
[SiW_11_O_39_][(CH_3_)_3_(C_8_H_17_)N]_8_	[SiW_11_O_39_][TMOA]_8_
[SiW_12_O_40_][(CH_3_)_3_(C_8_H_17_)N]_4_	[SiW_12_O_40_][TMOA]_4_

## Supplementary Materials

The followings are available online, Figure S1: Environmental scanning transmission electron microscopy (ESTEM) images of antifungal activity of the [SiW_11_O_39_][THTDA]_8_ and [SiW_12_O_40_][THTDA]_4_ POM-ILs on historical brick samples, taken at 642× and 3000× magnification. Table S1: Summary of the POM-ILs used in this work, their POM and ionic liquid precursors and the solvent used for their synthesis. Table S2: Thermogravimetric analysis of POM-ILs (MW = molecular weight). Table S3: Elemental analysis of POM-ILs. The values are given as mass percentages (%). Table S4: FT-IR analysis of POM-ILs (cm^−1^).

## References

[1] Guillitte O., Baer N.S., Fitz S., Livingston R.A. (1998). Bioreceptivity and biodeterioration of brick structures. Conservation of Historic Brick Structures.

[2] Gómez-Cornelio S., Mendoza-Vega J., Gaylarde C.C., Reyes-Estebanez M., Morón-Ríos A., De la Rosa-García S., Ortega-Morales B.O. (2012). Succession of fungi colonizing porous and compact limestone exposed to subtropical environments. Fungal Biol..

[3] Salvadori O., Municchia A.C. (2016). The Role of fungi and lichens in the biodeterioration of stone monuments. Open Conf. Proc. J..

[4] Cwalina B., Liengen T., Feron D., Basseguy R., Beech I.B. (2014). Biodeterioration of concrete, brick and other mineral-based building materials. Understanding Biocorrosion. Fundamentals and Applications.

[5] Sterflinger K. (2000). Fungi as geologic agents. Geomicrobiol. J..

[6] Abdel Ghany T.M., Omar A.M., Elwkeel F.M., Al Abboud M.A. (2019). Fungal deterioration of limestone false-door monument. Heliyon.

[7] Lan W., Li H., Wang W.D., Katayama Y., Gu J.D. (2010). Microbial community analysis of fresh and old microbial biofilms on Bayon temple sandstone of Angkor Thom, Cambodia. Microb. Ecol..

[8] Gu J.D., Kigawa R., Sato Y., Katayama Y. (2013). Addressing the microbiological problems of cultural property and archive documents after earthquake and tsunami. Int. Biodeterior. Biodegrad..

[9] Thrasher J.D. (2016). Fungi, bacteria, nano-particuletes, mycotoxins and human health in water-damaged indoor environments. J. Community Public Health Nurs..

[10] Järvi K., Hyvärinen A., Täubel M., Karvonen A.M., Turunen M., Jalkanen K., Patovirta R., Syrjänen T., Pirinen J., Salonen H. (2018). Microbial growth in building material samples and occupants’ health in severely moisture-damaged homes. Indoor Air.

[11] Baxi S.N., Portnoy J.M., Larenas-Linnemann D., Phipatanakul W., Barnes C., Baxi S., Grimes C., Horner W., Kennedy K., Levetin E.J. (2016). Exposure and health effects of fungi on humans. J. Allergy Clin. Immunol. Pract..

[12] Sharpe R.A., Bearman N., Thornton C.R., Husk K., Osborne N.J. (2015). Indoor fungal diversity and asthma: A meta-analysis and systematic review of risk factors. J. Allergy Clin. Immunol..

[13] Nevalainen A., Täubel M., Hyvärinen A. (2015). Indoor fungi: Companions and contaminants. Indoor Air.

[14] Nielsen K.F. (2003). Mycotoxin production by indoor mold. Fungal Genet. Biol..

[15] Salin J.T., Salkinoja-Salonen M., Salin P.J., Nelo K., Holma T., Ohtonen P., Syrjälä H. (2017). Building-related symptoms are linked to the in vitro toxicity of indoor dust and airborne microbial propagules in schools: A cross-sectional study. Environ. Res..

[16] Rylander R., Lin R. (2000). (1→3)-β-d-glucan—Relationship to indoor air-related symptoms, allergy and asthma. Toxicology.

[17] Gumerova N.I., Rompel A. (2020). Polyoxometalates in solution: Speciation under spotlight. Chem. Soc. Rev..

[18] Streb C. (2012). New trends in polyoxometalate photoredox chemistry: From photosensitisation to water oxidation catalysis. Dalton Trans..

[19] Yamase T. (2005). Anti-tumor, -viral, and -bacterial activities of polyoxometalates for realizing an inorganic drug. J. Mater. Chem..

[20] Bijelic A., Aureliano M., Rompel A. (2018). The antibacterial activity of polyoxometalates: Structures, antibiotic effects and future perspectives. Chem. Commun..

[21] Murugesan S., Quintero O.A., Chou B.P., Xiao P., Park K., Hall J.W., Jones R.A., Henkelman G., Goodenough J.B., Stevenson K.J. (2014). Wide electrochemical window ionic salt for use in electropositive metal electrodeposition and solid state Li-ion batteries. J. Mater. Chem. A.

[22] Mezzetta A., Perillo V., Guazzelli L., Chiappe C. (2019). Thermal behavior analysis as a valuable tool for comparing ionic liquids of different classes. J. Therm. Anal. Calorim..

[23] Aschenbrenner O., Supasitmongkol S., Taylor M., Styring P. (2009). Measurement of vapour pressures of ionic liquids and other low vapour pressure solvents. Green Chem..

[24] Herrmann S., De Matteis L., de la Fuente J.M., Mitchell S.G., Streb C. (2017). Removal of multiple contaminants from water by polyoxometalate supported ionic liquid phases (POM-SILPs). Angew. Chem. Int. Ed..

[25] Herrmann S., Kostrzewa M., Wierschem A., Streb C. (2014). Polyoxometalate ionic liquids as self-repairing acid-resistant corrosion protection. Angew. Chem. Int. Ed. Engl..

[26] Kubo A.-L., Kremer L., Herrmann S., Mitchell S.G., Bondarenko O.M., Kahru A., Streb C. (2017). Antimicrobial activity of polyoxometalate ionic liquids against clinically relevant pathogens. ChemPlusChem.

[27] Misra A., Castillo I.F., Müller D.P., González C., Eyssautier-Chuine S., Ziegler A., de la Fuente J.M., Mitchell S.G., Streb C. (2018). Polyoxometalate-ionic liquids (POM-ILs) as anticorrosion and antibacterial coatings for natural stones. Angew. Chem. Int. Ed..

[28] Misra A., Zambrzycki C., Kloker G., Kotyrba A., Anjass M.H., Franco Castillo I., Mitchell S.G., Güttel R., Streb C. (2020). water purification and microplastics removal using magnetic polyoxometalate-supported ionic liquid phases (magPOM-SILPs). Angew. Chem. Int. Ed. Engl..

[29] Rajkowska K., Otlewska A., Koziróg A., Piotrowska M., Nowicka-Krawczyk P., Hachułka M., Wolski G.J., Kunicka-Styczyńska A., Gutarowska B., Żydzik-Białek A. (2014). Assessment of biological colonization of historic buildings in the former Auschwitz II-Birkenau concentration camp. Ann. Microbiol..

[30] Kemme M., Heinzel-Wieland R. (2018). Quantitative assessment of antimicrobial activity of PLGA films loaded with 4-hexylresorcinol. J. Funct. Biomater..

[31] Hage-Hülsmann J., Grünberger A., Thies S., Santiago-Schübel B., Klein A.S., Pietruszka J., Binder D., Hilgers F., Domröse A., Drepper T. (2018). Natural biocide cocktails: Combinatorial antibiotic effects of prodigiosin and biosurfactants. PLoS ONE.

[32] Piotrowska M., Otlewska A., Rajkowska K., Koziróg A., Hachułka M., Nowicka-Krawczyk P., Wolski G.J., Gutarowska B., Kunicka-Styczyńska A., Zydzik-Białek A. (2014). Abiotic determinants of the historical buildings biodeterioration in the former Auschwitz II-Birkenau concentration and extermination camp. PLoS ONE.

[33] Gutarowska B., Żakowska Z. (2002). Elaboration and application of mathematical model for estimation of mould contamination of some building materials based on ergosterol content determination. Int. Biodeterior. Biodegrad..

[34] Kaminskyj S.G., Dahms T.E. (2008). High spatial resolution surface imaging and analysis of fungal cells using SEM and AFM. Micron.

[35] Petkovic M., Hartmann D.O., Adamova G., Seddon K.R., Rebeloa L.P.N., Pereira C.S. (2012). Unravelling the mechanism of toxicity of alkyltributylphosphonium chlorides in *Aspergillus nidulans* conidia. New J. Chem..

[36] Yoo B., Shah J.K., Zhua Y., Maginn E.J. (2014). Amphiphilic interactions of ionic liquids with lipid biomembranes: A molecular simulation study. Soft Matter.

[37] Egorova K.S., Gordeev E.G., Ananikov V.P. (2017). Biological activity of ionic liquids and their application in pharmaceutics and medicine. Chem. Rev..

[38] Koziróg A., Otlewska A., Gapińska M., Michlewska S. (2019). Influence of gemini surfactants on biochemical profile and ultrastructure of *Aspergillus brasiliensis*. Appl. Sci..

[39] Inoue M., Suzuki T., Fujita Y., Oda M., Matsumoto N., Yamase T. (2006). Enhancement of antibacterial activity of beta-lactam antibiotics by [P_2_W_18_O_62_]^6−^, [SiMo_12_O_40_]^4−^, and [PTi_2_W_10_O_40_]^7−^ against methicillin-resistant and vancomycin-resistant *Staphylococcus aureus*. J. Inorg. Biochem..

[40] Rajkowska K., Koziróg A., Otlewska A., Piotrowska M., Nowicka-Krawczyk P., Brycki B., Kunicka-Styczyńska A., Gutarowska B. (2016). Quaternary ammonium biocides as antimicrobial agents protecting historical wood and brick. Acta Biochim. Pol..

