# Supplementary material for: Antifungal Activity of Polyoxometalate-Ionic Liquids on Historical Brick
Source: Molecules. 2020 Dec 1;25(23):5663. doi: 10.3390/molecules25235663 (PMC7729500; doi:10.3390/molecules25235663)
Supplement: Supplementary file 1 [file molecules-25-05663-s001.pdf]

- Supporting Information -

Antifungal activity of polyoxometalate-ionic liquids on historical brick

Katarzyna Rajkowska,[1,*] Anna Koziróg,[1] Anna Otlewska,[1] Małgorzata Piotrowska,[1] Elena Atrián-Blasco,[2,3] Isabel Franco-Castillo[2,3] and Scott G. Mitchell*[2,3]

[1] Institute of Fermentation Technology and Microbiology, Lodz University of Technology, Wólczańska 171/173, 90-924 Lodz, Poland

[2] Instituto de Nanociencia y Materiales de Aragón (INMA), Consejo Superior de Investigaciones Científicas-Universidad de Zaragoza, 50009 Zaragoza, Spain

[3] CIBER-BBN, Instituto de Salud Carlos III, Madrid, Spain

* Correspondence: katarzyna.rajkowska@p.lodz.pl and scott.mitchell@csic.es

Contents

1. Instrumentation
2. Synthesis and characterization
3. Environmental scanning transmission electron microscopy (ESTEM) images
4. References

1. Instrumentation

Elemental Analysis: Elemental analyses (CHN) were performed by Interdepartmental Investigation Service (SIDI, Universidad Autónoma de Madrid) on a LECO CHNS-932 Elementary Chemical Analyzer.

FT-IR spectroscopy: FT-IR spectra were recorded on a Jasco FTIR 4100 spectrometer equipped with an ATR accessory. The samples were measured directly on the ATR, in a frequency range of 4000 – 600 cm^{-1} and a resolution of 2 cm^{-1}.

Thermogravimetric analysis: thermogravimetric analyses were recorded on a TGA Q5000 from TA Instruments in air.

2. Synthesis and characterization

For the synthesis of the eight different POM-ILs, three different Keggin-type POM precursors were used (see **Table S1**). Phosphotungstic acid, $H_3PW_{12}O_{40}$, and quaternary ammonium salts were purchased from Sigma Aldrich. $K_4SiW_{12}O_{40}$ and $K_8SiW_{11}O_{39}$ were prepared according to the procedure described in the literature.[1]

Table S1. Summary of the POM-ILs used in this work, their POM and ionic liquid precursors and the solvent used for their synthesis.

POM-IL	POM	Ionic Liquid	Solvent
$[PW_{12}O_{40}][THexA]_3$	$H_3PW_{12}O_{40}$	$[(C_6H_{13})_4N]Br$	dichloromethane
$[PW_{12}O_{40}][THepA]_3$	$H_3PW_{12}O_{40}$	$[(C_7H_{15})_4N]Br$	dichloromethane
$[PW_{12}O_{40}][TOctA]_3$	$H_3PW_{12}O_{40}$	$[(C_8H_{17})_4N]Br$	chloroform
$[SiW_{11}O_{39}][THTDA]_8$	$K_8[\alpha\text{-}SiW_{11}O_{39}]$	$[(C_6H_{13})_3(C_{14}H_{29})N]Br$	toluene
$[SiW_{12}O_{40}][THTDA]_4$	$K_4[\alpha\text{-}SiW_{12}O_{40}]$	$[(C_6H_{13})_3(C_{14}H_{29})N]Br$	chloroform
$[SiW_{11}O_{39}][TOctA]_8$	$K_8[\alpha\text{-}SiW_{11}O_{39}]$	$[(C_8H_{17})_4N]Br$	dichloromethane
$[SiW_{11}O_{39}][TMOA]_8$	$K_8[\alpha\text{-}SiW_{11}O_{39}]$	$[(CH_3)_3(C_8H_{17})N]Br$	dichloromethane
$[SiW_{12}O_{40}][TMOA]_4$	$K_4[\alpha\text{-}SiW_{12}O_{40}]$	$[(CH_3)_3(C_8H_{17})N]Br$	dichloromethane

General procedure for the synthesis of POM-ILs

A desired amount of POM was dissolved in 50 mL of pre-heated distilled water at 50 °C. The mixture was stirred until complete dissolution. In a beaker, the appropriate amount of the corresponding ionic liquid was dissolved in 80 mL of the solvent (see Table S1) and stirred until complete dissolution. Then, the POM solution was slowly added to the IL solution and the mixture was stirred for 3 h. After this time, the mixture was poured into a separation funnel and once the two phases are clearly separated, the organic phase –

including the POM-IL – is extracted into a round bottom flask. The organic solvent was removed in a rotatory evaporator, and the product was isolated.

Table S2. Thermogravimetric analysis of POM-ILs (MW = molecular weight).

POM-IL	MW (POM)	MW (IL) x n	MW (POM-IL)	% m IL, calculated	% m IL, experimental
$[PW_{12}O_{40}][THexA]_3$	2877.03	1064.02	3941.05	27.00	30.70
$[PW_{12}O_{40}][THepA]_3$	2877.03	1232.34	4109.37	29.99	32.67
$[PW_{12}O_{40}][TOctA]_3$	2877.03	1400.66	4277.69	32.74	33.15
$[SiW_{11}O_{39}][THTDA]_8$	2674.30	3735.09	6409.39	58.28	57.64
$[SiW_{12}O_{40}][THTDA]_4$	2874.14	1867.54	4741.68	39.39	38.30
$[SiW_{11}O_{39}][TOA]_8$	2674.30	3735.09	6409.39	58.28	59.10
$[SiW_{11}O_{39}][TMOA]_8$	2674.30	1378.64	4052.94	34.02	31.89
$[SiW_{12}O_{40}][TMOA]_4$	2874.14	689.32	3563.46	19.34	25.88

Table S3. Elemental analysis of POM-ILs. The values are given as mass percentages (%).

	calculated			experimental		
POM-IL	C	H	N	C	H	N
$[PW_{12}O_{40}][THexA]_3$	21.94	3.99	1.07	24.30	4.30	1.20
$[PW_{12}O_{40}][THepA]_3$	24.55	4.41	1.02	26.50	4.60	1.10
$[PW_{12}O_{40}][TOctA]_3$	26.95	4.81	0.98	27.55	4.72	0.96
$[SiW_{11}O_{39}][THTDA]_8$	47.97	8.55	1.75	48.16	8.59	2.02
$[SiW_{12}O_{40}][THTDA]_4$	32.42	5.78	1.18	41.00	7.00	1.40
$[SiW_{11}O_{39}][TOA]_8$	47.97	8.55	1.75	48.09	8.76	1.89
$[SiW_{11}O_{39}][TMOA]_8$	26.08	5.17	2.76	20.88	4.34	2.03
$[SiW_{12}O_{40}][TMOA]_4$	14.83	2.94	1.57	16.22	3.60	1.64

Table S4. FT-IR analysis of POM-ILs (cm^{-1}).

POM-IL	ν P-O / Si-O	ν W=O	ν, δ W-O-W
$[PW_{12}O_{40}][THexA]_3$	1078 (s)	974 (s)	893 (s), 801 (vs), 727 (w)
$[PW_{12}O_{40}][THepA]_3$	1077 (s)	977 (s)	890 (s), 800 (vs), 726 (w)
$[PW_{12}O_{40}][TOctA]_3$	1079 (s)	974 (s)	896 (s), 813 (vs), 724 (w)
$[SiW_{11}O_{39}][THTDA]_8$	1081 (w)	993 (w), 953 (s)	897 (s), 813 (w), 750 (s), 693 (s), 657 (s)
$[SiW_{12}O_{40}][THTDA]_4$	1080 (w)	965 (s)	951 (w), 917 (s), 844 (m), 792 (s), 729 (m)
$[SiW_{11}O_{39}][TOA]_8$	1075 (w)	997 (m), 954 (m)	900 (s), 807 (s), 532 (m)
$[SiW_{11}O_{39}][TMOA]_8$	1073 (w)	978 (s), 936 (s)	861 (vs), 787 (vs), 722 (vs), 670 (s), 632 (s)
$[SiW_{12}O_{40}][TMOA]_4$	1080 (w)	988 (s)	935 (s), 870 (sh), 861 (vs), 788

			(vs), 711 (vs), 655 (w), 649 (w), 640 (w), 626 (w)

3. Environmental scanning transmission electron microscopy (ESTEM)

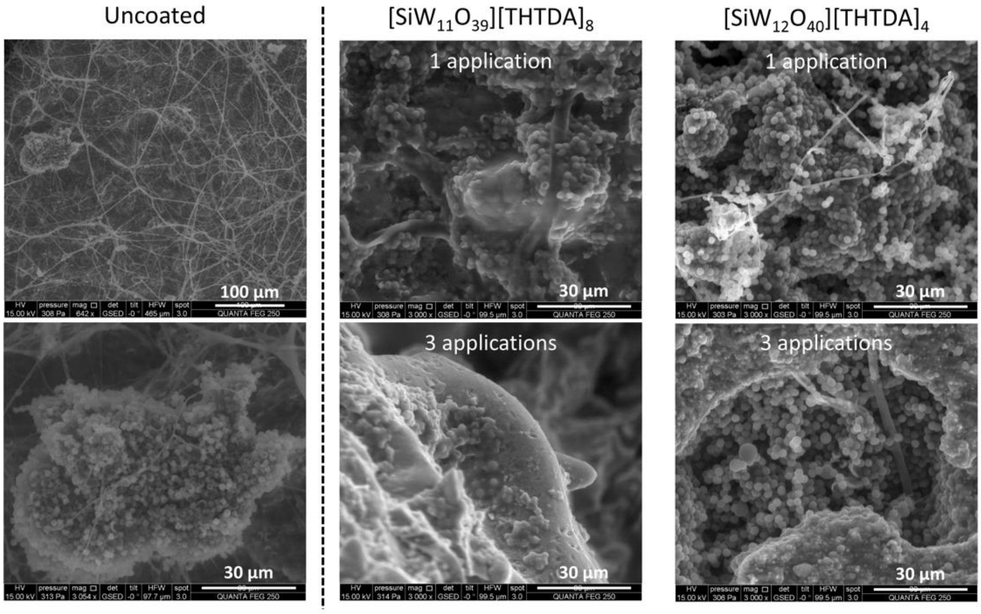

Figure S1. Environmental scanning transmission electron microscopy (ESTEM) images of antifungal activity of the $[SiW_{11}O_{39}][THTDA]_8$ and $[SiW_{12}O_{40}][THTDA]_4$ POM-ILs on historical brick samples, taken at 642 x and 3000 x magnification.

4. References

1. Tézé, A.; Hervé, G.; Finke, R.G.; Lyon, D.K. α-, β-, and γ-Dodecatungstosilicic Acids: Isomers and Related Lacunary Compounds. *Inorganic Syntheses*; 1990; pp. 85–96, Online ISBN: 9780470132586; Print ISBN: 9780471509769.
